# Clinical benefits of FOLFOXIRI combined with bevacizumab for advanced-stage primary signet ring cell carcinoma of the appendix: A case report

**DOI:** 10.1097/MD.0000000000034412

**Published:** 2023-08-04

**Authors:** Nana Huang, Yishan Lu, Rui Wang, Ping Gao, Ge Liu

**Affiliations:** a The Second Ward of Gastroenterology, Cancer Hospital of Liaoning Provience; b Department of Dermatology, The First Affiliated Hospital of Dalian Medical University, Dalian, China; c Department of Pancreatic Surgery, West China Hospital, Sichuan University, Chengdu, China; d Department of Oncology, The First Affiliated Hospital of Dalian Medical University, Dalian, China; e Department of Anorectal Surgery, The First Affiliated Hospital of Dalian Medical University, Dalian, China.

**Keywords:** bevacizumab, case report, FOLFOXIRI, signet ring cell carcinoma, XELOX

## Abstract

Signet-ring cell carcinoma, which is an infrequent type of colorectal cancer. Abdominal pain is the primary presenting complaint of patients with acute appendicitis. It is difficult to diagnose patients with appendiceal carcinomas accompanying with symptoms of acute appendicitis.

**Patient Concerns:**

A 33-year-old female patient was admitted to our hospital, with chief complaints of “bilateral pelvic space-occupying lesions for 1 month, aggravated abdominal distension, and she accompanied with diarrhea for 3 days.”

**Diagnosis:**

The patient was with primary signet ring cell carcinoma of the appendix, presented with acute appendicitis, as well as bilateral ovarian metastasis and peritoneal implantation metastasis.

**Interventions:**

She was then treated with irinotecan, oxaliplatin, calcium folinate, 5-FU combined with bevacizumab, surgical treatment, and postoperative adjuvant treatment with oxaliplatin, capecitabine regimen to consolidate the efficacy.

**Outcomes:**

The patient is in good conditions, and postoperative adjuvant chemotherapy is in progress as well.

**Conclusion:**

The outcomes highlighted the importance of strict histopathologic assessment for appendiceal adenocarcinoma, and provided new ideas for the diagnosis and treatment of advanced-stage signet ring cell carcinoma of the appendix.

## 1. Introduction

Primary tumors of the appendix represent approximately 0.4% of all gastrointestinal tract tumors, and are mainly found in about 1% of appendectomies.^[1]^ The most frequent location of tumors with the signet ring cell pattern is the stomach (96%), followed by the colon, rectum, gallbladder, and pancreas. Signet-ring cell carcinoma (SRCC), which is an infrequent type of colorectal cancer, accounts for 0.5% to 2.6% of all adenocarcinomas.^[2]^ Abdominal pain is the primary presenting complaint of patients with acute appendicitis. It is difficult to diagnose patients with appendiceal carcinomas accompanying with symptoms of acute appendicitis.^[3]^ For the treatment of signet ring cell carcinoma of the appendix (SRCC-A), surgical treatment is clinically significant. At present, the majority of SRCC-A patients are treated with right hemicolectomy, and postoperative adjuvant chemotherapy can further improve the prognosis of such patients.^[4]^ FOLFOX (oxaliplatin plus leucovorin and infusional FU) with or without bevacizumab were used clinically. The common organ for metastases are ovaries, women who underwent resection of ovarian metastases had a 31% 5-year survival rate.^[5]^ We presented a case of advanced stage primary signet-ring cell carcinoma of the appendix complicated with bilateral ovarian metastases and peritoneal implantation metastases, which was proved to be clinically beneficial after irinotecan, oxaliplatin, calcium folinate, 5-FU (FOLFOXIRI) preoperative regimen combined with bevacizumab as neoadjuvant systemic therapy. In addition, this paper reviews the literature on SRCC-A.

## 2. Case presentation

### 2.1. Chief complaints

A 33-year-old female patient was admitted to our hospital on July 10, 2020, due with chief complaints of “bilateral pelvic space-occupying lesions for 1 month, aggravated abdominal distension, and she accompanied with diarrhea for 3 days.”

### 2.2. History of present illness

Abdominal ultrasound that was conducted in another hospital revealed bilateral pelvic space-occupying. However, no abnormalities were observed through routine gynecological ultrasound examination performed when using contraceptive ring 1 year before her admission.

### 2.3. History of past illness

She had undergone induced abortion and cesarean section, and she also had a history of intrauterine device placement. There was no family history of ovarian or gastrointestinal malignancy.

### 2.4. Physical examination

Abdominal and gynecological ultrasound did not show any abnormal finding 1 year ago, the mass is now up to 10 cm on the left side and 6 cm on the right side, accompanied by peritoneal effusion which is newly developed, with increased tumor markers, and rapid disease progression, alignant tumor is considered.

### 2.5. Laboratory examinations

Prealbumin level was 145 mg/L, serum albumin level was 32.7 g/L, and total protein concentration was 53.4 g/L. Protein quantification was 35.9 g/L, adenosine deaminase level is 3 U/L, and lactate dehydrogenase level was 99 U/L; On urinalysis, urine specific gravity was 1.024, weakly positive for protein in the urine. The total cell numbers were 1420/μL, and the proportion of lymphocytes was 62%.

### 2.6. Imaging examinations

The abdominal distension showed a huge mass with the size of 10 × 10 cm in pelvic cavity, and the other palpation was unclear. The levels of tumor markers are listed in Table 1.

**Table 1:** Changes of tumor markers at different stages.

TM	BC	AC	BO	AO	NL
CEA	7.54 ng/mL	24.47 ng/mL	20.08 ng/mL		0–5 ng/mL
CA19–9	<0.600 U/mL	<0.600 U/mL	<0.600 U/mL		0–27 U/mL
CA125	136.6 U/mL	16.85 U/mL	12.7 U/mL		0–35 U/mL
AFP	0.65 U/mL		0.87 U/mL		0–5.8 IU/mL
HE4	77.76 pmol/L	57.26 pmol/L			0–60.5 pmol/L
SCC	4.20 ng/mL				0–2.5 ng/mL
NSE	7.68 ng/mL				0–24 ng/mL

AC = after chemotherapy, AO = after operation, BC = before chemotherapy, BO = before operation, NL = normal level.

### 2.7. Further diagnostic work-up

**Figure 1.**
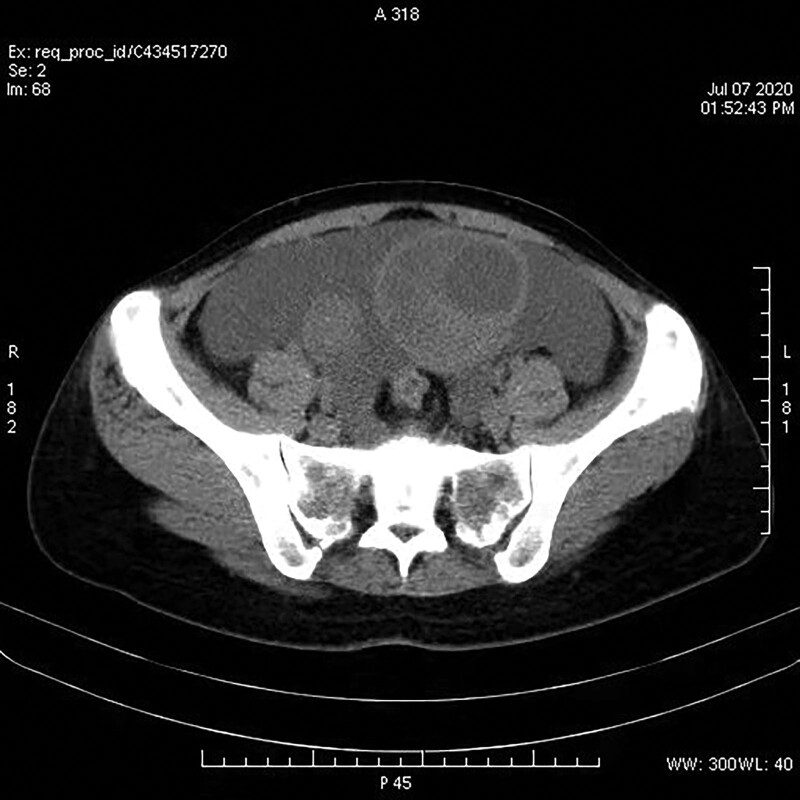
The pelvic cavity showed circular mass with smooth boundary, with a range of about 10.5 × 8.4 cm.

Combined with the patient symptoms and the results of laboratory examinations, cancerous ascites were considered. Computed tomography (CT) scan images of the whole abdomen are showed in Figure 1. A quasi-circular space-occupying shadow was found in pelvic cavity with a smooth boundary, with a range of about 10.5 × 8.4 cm for the larger one, and a quasi-circular low-density shadow was observed as well. No clear signs of invasion were found in the rectum, and the surrounding tissues of the mass were moved. Pelvic magnetic resonance imaging was carried out. It showed space occupation in bilateral adnexal areas, as cystic ovarian carcinoma could not be ruled out. Abdominal ultrasound showed a solid space occupying in liver (with high possibility of hemangioma). No obvious abnormalities were observed in gallbladder, pancreas, spleen, kidneys, and ureters.

Gastroscopy showed chronic non-atrophic gastritis. Enteroscopy revealed that the ileocecal valve was shaped. There was a pattern of protruding lesions in the appendix, and its size was about 1.5 cm. The surface was lobulated, erosive, and disordered neoplastic blood vessels were extremely visible.

**Figure 2.**
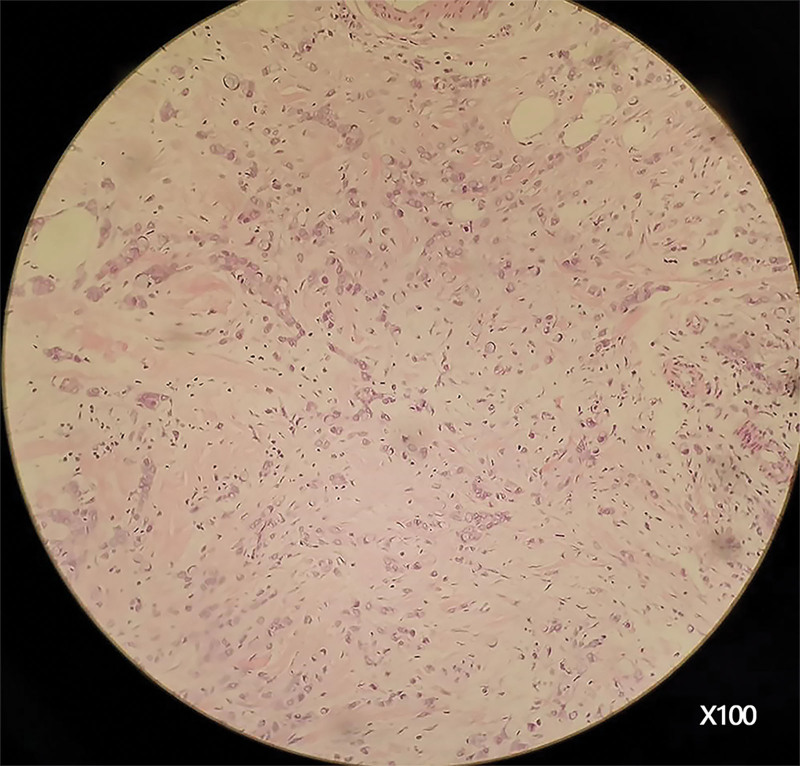
Pathology under colonoscopy: a large number of abnormally proliferative cells were found to replace part of the normal appendix, and the nuclei were squeezed by mucus to one side in a ring shape.

The results of colonoscopy and pathological examination are shown in Figure 2. The colonoscopy showed that ascending colon, descending colon, sigmoid colon, and rectum were normal, without hyperemia, erosion, ulceration, and abnormal swelling. Under the microscope, a large number of abnormally proliferative cells were found in appendix tissue, which were diffusely distributed. The cells contained rich mucus, with the nucleus pressed to one side of the cell and formation of ring-shaped. There were also atypical cells with large nuclei, deep staining, acidophilic cytoplasm and obvious atypia. Pathologic diagnosis of mucosal biopsy material was signet ring cell carcinoma (Appendiceal crypt).

Immunohistochemical examination of MLH 1, MSH 2, MSH 6, and PMS 2 was performed, and the results were stated as MLH 1, MSH 2, MSH 6 complete diffuse lack of nuclear reactions as positive microsatellite instability MSI event, and PMS 2 was partly positive.

### 2.8. Multidisciplinary expert consultation

*Georgios Gatzounis, MD, PhD, Professor and Chief, Department of Neurosurgery, University of Patras Medical School.*

*Ping Gao, Master of Oncology, Chief Physician, Postgraduate Supervisor, Department of Oncology, The First Affiliated Hospital of Dalian Medical University.*

From the personal medical history, abdominal and gynecological ultrasound did not show any abnormal finding 1 year ago, the mass is now up to 10 cm on the left side and 6 cm on the right side, accompanied by peritoneal effusion which is newly developed, with increased tumor markers, and rapid disease progression, alignant tumor is considered.

*Ge Liu, Director of the First Department of General Surgery, Director of Anorectal Surgery, MD, Professor, Master Supervisor, Department of Anorectal Surgery, The First Affiliated Hospital of Dalian Medical University.*

From the personal medical history, abdominal and gynecological ultrasound did not show any abnormal finding 1 year ago, the mass is now up to 10 cm on the left side and 6 cm on the right side, accompanied by peritoneal effusion which is newly developed, with increased tumor markers, and rapid disease progression, alignant tumor is considered.

### 2.9. Final diagnosis

Based on the results of colonoscopy and related auxiliary examinations, the patient was diagnosed as SRCC-A, implantation metastasis of ovarian cancer, and abdominal effusion.

The final diagnosis of the presented case is spontaneous cerebral abscess due to B. subtilis.

### 2.10. Treatment

Based on the results of colonoscopy and related auxiliary examinations, the patient was diagnosed as SRCC-A, implantation metastasis of ovarian cancer, and abdominal effusion.

**Figure 3.**
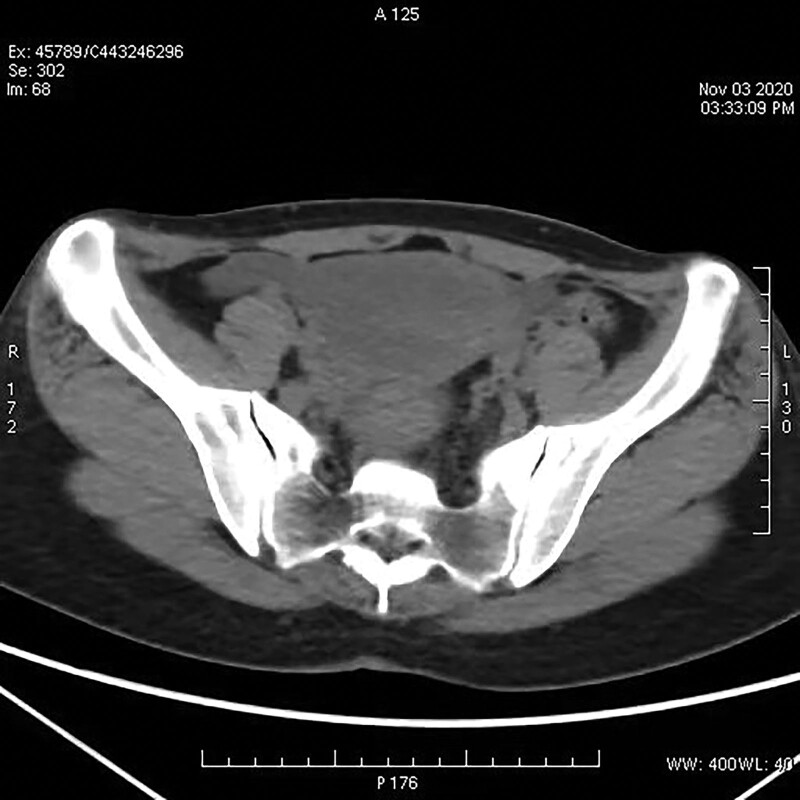
CT scan of the abdomen shows a pelvic mass with a malignant tendency of mass, smaller than before.

The patient had advanced stage malignant tumor, large tumor volume, intraperitoneal metastasis, and poor prognosis. Therefore, chemotherapy was considered in in medical oncology. The further treatment plan was decided in accordance with its efficacy. FOLFOXIRI regimen (irinotecan 165 mg/m^2^, intravenous infusion, oxaliplatin 85 mg/m^2^, calcium linovinate 200 mg/m^2^, 5-FU 3200 mg/m^2^, continuous intravenous infusion for 48 h, 14 days as 1 cycle) combined with bevacizumab was selected on the basis of the potential efficacy as well as the confirmed safety. After 5 cycles of chemotherapy, tumor markers were significantly reduced, as shown in Table 1. Reexamination of abdominal CT showed pelvic mass with malignant tendency of mass occupying. An abdominal CT scan revealed a pelvic mass occupying the pelvic cavity on less than before. After surgical consultation, the patient condition was improved, local symptoms were significantly relieved, abdominal effusion was basically absorbed, pelvic effusion was less than before. Surgical indications were shown in Figure 3.

Surgical treatment was performed in the Department of Anorectal Surgery of our hospital. Preoperative levels of tumor markers are shown in Table 1. Repeated endoscopy after chemotherapy revealed change of periappendiceal tumor. Further investigation was needed for submucosal bulging lesion. Pathology analyses of random mucosal biopsies revealed chronic inflammatory changes. Makd full preoperative preparations, the patient underwent laparoscopic hysterectomy with bilateral adnexectomy, palliative ileocecal resection and end-to-side anastomosis of the distal ileum ascending colon were performed. Operative findings were grayish white nodules scattered on the parietal peritoneum, the visceral peritoneum, and mesentery, which ranged from military to rice grain-sized. Implantation was considered. The appendix was invaded by the tumor and became thickened and hard, without adhesion to the surrounding tissues. Considering extensive abdominal metastasis, radical surgery could not be performed. After consultation with the patient and her family, laparoscopic hysterectomy and bilateral adnexectomy, palliative ileocecal resection, and end-to-side anastomosis of the distal ileum ascending colon were conducted.

### 2.11. Outcome and follow-up

Postoperative pathology showed that cecum signet ring cell appeared in appendix and appendicular fossa, with the tubular glands and eosinophil infiltration, the cancer cell nests in the appendix, the surrounding adipose tissue beyond the muscular layer, and peritoneal myxoma. Cancer cells observed in the mucosa lamina propria, including arterioles and veins, tumor emboli within vascular channels were detected, surface mucous membranes remaining intact, neural invasion was visible. Therefore, a close clinical examination is recommended to rule out the gastrointestinal tract as the origins of the metastasis.

Immunohistochemical results were Syn (part +), CD56 (+), CDX2 (+), CgA (part +), cytokeratin 20 (CK20) (+), CK7 (−), Ki67 (Li: 10%), CK (+). In addition, special stain PAS was positive. Eosinophilic cytoplasm and signet ring cell infiltration on ovaries were present. Therefore, metastatic signet ring cell carcinoma should be considered. Metastatic signet ring cell carcinoma was seen on 1 fallopian tube (there is a vesicular appendage on this side), while no metastases were observed on the other side. Immunohistochemical results were CD56 (+), CDX2 (+), CgA (−), CK20 (+), CK7 −), Ki 67 (Li: 10%), Syn (part +). Special stain PAS was positive. Endometrium-serosal full-layer SRCC infiltration was observed, infiltration of a signet ring cell carcinoma into the mucosa of the cervix and cervical canal to the adventitia was observed, which was considered of metastasis. The lymph nodes submitted for examination showed metastatic carcinoma (3/4, 1/1). No metastatic carcinoma was observed in lymph nodes in free adipose tissue and lymph nodes (201 group) (0/1, 0/6), and SRCC was observed in peripheral fat of lymph nodes in 201 group. The patient recovered smoothly and was discharged on the 8th day after surgery. Postoperative adjuvant chemotherapy was performed in the department of oncology 1 month after surgery, using oxaliplatin, capecitabine (XELOX) (oxaliplatin 130 mg/m^2^ intravenous infusion for 2 h d1, capecitabine 1800 mg/m^2^/D, bid d1–14, q21d) combined with bevacizumab. At present, the patient is in good conditions, and postoperative adjuvant chemotherapy is in progress as well.

## 3. Discussion

The Fifth Edition of the World Health Organization classification divided appendiceal tumors into the following categories: serrated lesions and polyps; mucinous tumors; adenocarcinoma; neuroendocrine tumors.^[2,6]^ SRCC-A is a rare and unique subtype of adenocarcinoma of the appendix. The histopathology result shows a large amount of mucus in the cell cytoplasm, pushing the nucleus to one side. The abundant intracytoplasmic mucus pushes the nucleus to at least 50% of the tumor volume. The incidence of this condition is approximately 0.15 per million per year.^[4]^ The mean age for this specific type of appendiceal carcinoma is 58.8 years old,^[2]^ which is younger than other subtypes. In addition, the incidence of signet ring cell subtypes is stably increasing, which may be attributed in part to enhanced awareness of the disease process and improved techniques, standardized diagnostic algorithms.^[7]^

Compared with the most common signet ring cell carcinoma of the stomach, a higher proportion of SRCC-A, colon and rectum is found in women, while there are also reports on no significant difference in incidence between men and women.^[2]^ Literature evidence reports that there is a higher prevalence of it in white people than in other races and is more common in elderly women, whereas race has no significant effect on survival rate.^[1,2]^ Among several subtypes of appendiceal tumors, SRCC has the worst prognosis, 60% of the tumors were histologically confirmed to be poorly differentiated or undifferentiated tumors of high grade.^[1]^ The median survival time was 26.0 months, factors to consider include age and the range of disease signet ring cell carcinoma studied in a prior investigation. SRCC is a subtype of carcinoma with unique clinical characteristics and poor survival rates.

Similar to mucinous adenocarcinoma, SRCC-A may have distant metastases at diagnosis, and 64% of patients have lymph node metastasis, which is significantly higher than that of other histological types.^[1,2]^ Zheng et al confirmed that SRCC-A is a distant tumor, accounting for 66.5% of the total cases.^[8]^ Ovary and peritoneum are common sites of metastasis, and Kulkarni et al concentrated on the ovarian implantation metastasis and extensive peritoneum metastasis.^[9]^ Although the peritoneal metastasis rate of SRCC-A is noticeable, it is rarely transferred to the liver and/or lung through blood, and the metastasis rate of liver and lung is low.^[7,10]^ The CT of chest and abdominal ultrasound confirmed that there was no liver or lung metastasis. To date, a limited number of cases of rare sites of metastasis have been reported.^[11]^

Primary SRCC-A lacks specific clinical manifestations. In contrast to other appendiceal neoplasms, which are often asymptomatic, the majority of patients with SRCC-A present with a sign of acute appendicitis.^[12]^ In addition, adenocarcinoma of the appendix is the most common gastrointestinal malignancy associated with perforation.^[5]^ The obstruction of intestinal lumen may make patients accompany with intestinal obstruction, according to a case reported by Suzuki et al^[13]^ Some cases were diagnosed with inflammatory bowel disease.^[14]^ Vukovic et al reported a case of Crohn disease who underwent right hemicolectomy plus lymph node biopsy. However, the definitive diagnosis was SRCC-A.^[15]^ This also indicated that caution must be taken to avoid misdiagnosis. Up to 40% of cases present with distant metastases to the peritoneum, liver, and/or ovaries.^[16–19]^ Kulkarni et al described a case who was misdiagnosed with ovarian torsion.^[9]^

In addition, SRCC-A lacks imaging manifestations, which may lead to misdiagnosis or missed diagnosis. Therefore, in the case of appendicitis, the pathology of intraoperative freezing should be used to rule out atypical lesions, so as to reduce the risk of reoperation. Imaging findings showed 5 signs of acute appendicitis, including appendiceal wall with thickness of >2 mm, abscess, periappendicitis, and appendicolith.^[20]^ Severe periappendiceal fat infiltration or periappendiceal abscess is indicative of perforation. The ultrasonographic manifestations of non-mucinous carcinoma of the appendix (including adenocarcinoma and SRCC) are relatively small intestinal lumen diameter, appendix wall thickening or submucosal hypoecho.^[21]^ A previous research confirmed that colon cancer presents the following features in high-resolution ultrasound: heterogeneous hypoechoic masses, irregular wall thickening, and absence of lamellar walls.^[21]^ Cho et al described the distinction between neoplastic and non-neoplastic inflammatory responses of the appendix. Firstly, the true lumen diameter of neoplastic inflammation of the appendix is typically smaller than that of acute appendicitis. In contrast to the centri-centric wall thickening observed in acute appendicitis, marginal wall thickening is a prominent feature of appendiceal neoplastic inflammatory response. Secondly, the submucosal and muscular layers are in form of monolayer, with a uniform and obvious hypoechoic texture, and the parietal layer disappears. Thirdly, the thickness of the appendix wall is significantly higher than that of the fat infiltration around the appendix.^[22]^

Pathological examination is highly essential for a definite diagnosis. Microscopic characteristics of SRCC includes a large number of abnormally proliferative cells evolving into appendiceal tissues with a diffuse distribution. The cells are rich in mucus, and the nucleus is squeezed by mucus to one side in a ring shape. There are also atypical cells with a large nucleus, deep staining, an acidophilic cytoplasm, and an obvious atypia. According to the diagnostic criteria of primary adenocarcinoma of the appendix, the diagnosis of primary SRCC-A should meet the following conditions: tumor infiltration and the mucosal muscular layer, mainly accompanying with the wall of the appendix, as well as various degrees of atrophic lymphoid tissue; SRCC can be diagnosed when the proportion of signet-ring cells in the tumor is >50%; Adenocarcinoma components with different degrees of differentiation can be mixed simultaneously in tumor tissues; The metastasis of signet ring cell carcinoma of stomach, intestinal tract, breast, prostate and bladder to appendix was ruled out by gastroenteroscopy, abdominal pelvic CT, and color Doppler ultrasound; goblet cell adenocarcinoma was excluded by immunohistochemical staining.^[23]^

As primary SRCC-A is extremely rare, its diagnosis needs to its differentiation from metastatic diseases. The most common site of metastasis is the stomach. Gastroenteroscopy is of great significance for the differential diagnosis. The results of gastroenteroscopy in the present case showed that and mucosa of cecum, ascending colon, transverse colon, descending colon, sigmoid colon, and rectum were normal, and there was no hyperemia and dissipation, ulcer or abnormal bulge, which ruled out the possibility that SRCC of stomach and intestinal tract would be metastasized to appendix. In addition, the diagnosis of SRCC-A is mainly accompanied with distant metastasis, and ovary is a common site of metastasis, thus, the appendiceal origin should be considered in patients with ovarian tumors. However, primary ovarian tumors can also metastasize to the appendix, and the identification of the source of ovarian tumors is highly significant for the diagnosis and treatment of patients. Immunohistochemical staining is of great significant to the differential diagnosis. Suzuki et al showed that CK20 was expressed only in epithelium of the gastrointestinal tract and urethral epithelium, while CK7 is expressed in ovarian tissues.^[13]^ Ronnett et al found that primary ovarian mucinous tumors express CK7, rather than CK20, while patients with appendiceal malignancy are diagnosed with CK20-positive, and CK7 is expressed in 50% cases. In the present case, immunohistochemical staining revealed CK7-negative and CK20-positive, the high expression of CK20 indicated that the primary lesion of the ovarian tumor was located in the malignant tumor of the appendix. Furthermore, SATB2 is a significant marker for determining the primary sites of MKTs of the ovary. It has been suggested that SATB2 is a useful marker for determining the primary sites of metastatic ovarian tumors.^[24]^ Studies have shown that CDX2 and SATB2 were positively expressed in all the lower gastrointestinal primary tumors and negatively expressed in the majority of upper gastrointestinal primary tumors.^[21]^ In addition, mucins (MUC-1, MUC-2) are helpful for the differentiation of metastatic appendix originated from the ovary. Yajima et al reported that all malignant tumors of the appendix were expressed with MUC-2, whereas ovarian cancer only expressed with MUC-1, rather than with MUC-2.^[20]^ Primary SRCC-A also needs to be differentiated from goblet cell adenocarcinoma, which has a unique growth pattern and is located in the submucosa. Goblet cell adenocarcinoma often infiltrates the appendix wall in a typical centripetal growth pattern and forms ill-defined masses. The signet ring-like cells are arranged in small, round, nest-like structures similar to normal intestinal epithelial cells. Immunohistochemical staining of signet ring-like cells in goblet cell adenocarcinoma was positive for CgA, SyN, and CD56.^[25]^

The optimal treatment for SRCC-A remains controversial. It has been suggested that right hemicolectomy should be performed for all non-cancerous appendicitis tumors (e.g., mucinous adenocarcinoma, goblet cell adenocarcinoma, signet ring cell carcinoma).^[4]^ Due to the difficulty of preoperative diagnosis, patients mainly rely on postoperative pathology for a more reliable diagnosis after primary simple appendectomy, which increases the risk of reoperation. For instance, Kulkarni et al reported a 45-year-old female patient who was misdiagnosed as ovarian torsion due to persistent abdominal pain in the right lower abdomen. She underwent appendectomy plus unilateral salping-tubal oophorectomy, and her right colon was resected for the second time after postoperative pathology, which confirmed SRCC-A.^[9]^ Similarly, Cho et al also reported a patient who received appendectomy and right salpingo-oophorectomy because of suspected acute appendicitis and ovarian tumor. The pathological examination found that the appendix wall was asymmetrically thickened due to signet ring cell infiltration, and the patient underwent right hemicolectomy.^[22]^

Tumor reduction surgery and intraperitoneal thermochemotherapy (CRS/HIPEC) are considered as the standard treatment for peritoneal dissemination of appendiceal cancer.^[1]^ An aggressive approach to these patients that includes CRS/HIPEC is advocated because of the possibility of long-term relapse-free survival (and possibly cure) in a subset of patients. Patients with undifferentiated adenocarcinoma with lymph node involvement and severe submucosal infiltration should consider secondary partial colectomy with lymph node dissection.^[26]^ Bertuzzo et al pointed out that right hemicolectomy should be performed for non-cancer-like appendiceal tumors with lymph node metastasis and mesoappendiceal penetration. In the event of peritoneal implantation metastasis, cytopenia and peritoneal hyperthermia should be performed. However, the 5-year survival rate was 68% for patients who received right hemicolectomy, and 20% for patients who received appendectomy alone.^[27]^ Data also show that the 5-year survival rate of subtotal colectomy is 26.5%, and that of partial colectomy (including appendectomy) is 20.9%.^[1]^ Therefore, a number of scholars suggested that all appendiceal masses should be treated with right hemicolectomy due to the invasive nature of the lesions, and patients who chose combined chemotherapy had a better prognosis than those undergoing surgery alone.^[4]^

Postoperative adjuvant therapy can reduce the recurrence rate, improve the survival rate and improve the prognosis. However, due to the lack of relevant research data, the treatment of SRCC-A is mainly inferred based on the data of colorectal cancer, and adjuvant treatment based on 5-FU chemotherapy is preferred, and the majority of patients adopt FOLFOX regimen, or in combination with bevacizumab based on data demonstrated therapeutic efficacy for metastatic colon cancer.^[7,14,28,29]^ Bevacizumab is an inhibitor of angiogenesis specific to vascular endothelial growth factor (VEGF). Bevacizumab binds to VEGF and prevents VEGF from interacting with its benefits on the endothelial cell surface. The United States Food and Drug Administration approved bevacizumab in combination with intravenous 5-fluorouracil for first- or second- line treatment in patients with metastatic colorectal cancer; The combination of fluorouracil-Irinotecan or fluorouracil-oxaliplatin is used as second-line therapy in patients with metastatic colorectal cancer whose disease is still progressing after previous first-line treatments with bevacizumab. For instance, Powell Ed et al reported a case of postoperative FOLFOX combined with bevacizumab that showed clinical benefit and sustained disease stability.^[30]^ Suzuki et al reported a case of peritoneal surface metastasis caused by primary appendix treated with 5-Fu and mitomycin intraperitoneally.^[13]^ In this case, the patient had advanced primary SRCC-A and was treated with FOLFOXIRI regimen combined with bevacizumab for 5 cycles, accompanying with a good curative effect. Relevant auxiliary examinations confirmed that the appendiceal mass disappeared, the mucosal biopsy tissue (appendiceal cryptus) showed chronic inflammatory changes, the pelvic mass was smaller than before, the local symptoms were significantly relieved, the abdominal effusion was basically absorbed. The patient had surgical indications and was subsequently treated surgically. At the same time, the postoperative recovery was good, and XELOX regimen was actively used to consolidate the efficacy of adjuvant chemotherapy. At present, adjuvant chemotherapy for SRCC-A is mostly reported in individual cases. According to the available data, this is the first reported case of advanced primary signet-ring cell carcinoma of the appendix with clear preoperative and pathological diagnosis, and after receiving FOLFOXIRI combined with bevacizumab, the tumor disappeared in the appendiceal crytum with surgical indications. Our case report can provide reference for the diagnosis and treatment of this disease. We recommend hemicolectomy after neoadjuvant therapy if the preoperative diagnosis of SRCC-A or lesion limitations is clear. The chemotherapy regimen can refer to the first-line treatment of advanced colorectal cancer and select irinotecan or oxaliplatin based combination therapy, such as FOLFOX (fluorouracil, leucovorin, and oxaliplatin), FOLFIRI (fluorouracil, leucovorin, and irinotecan) and FOLFOXIRI (fluorouracil, leucovorin, oxaliplatin, and irinotecan). Meanwhile, studies have shown that bevacizumab targeting VEGF can inhibit endothelial cell proliferation and new angiogenesis, and delay tumor growth and metastasis. Therefore, bevacizumab combined with chemotherapy is more advantageous than chemotherapy alone. In addition, the TRIBE study further compared the efficacy of FOLFOXIRI combined with bevacizumab and FOLFIRI combined with bevacizumab in the treatment of unresectable metastatic colorectal cancer, and the results showed that FOLFOXIRI combined with bevacizumab significantly prolonged the median PFS and significantly improved the disease control rate.^[31]^ If there is a late stage of distant metastasis at the time of diagnosis, postoperative adjuvant chemotherapy can also benefit patients. For example, the capecitabine-based XELOX regimen has been recognized for the treatment of metastatic colorectal cancer with low toxicity and good safety.

Among several subtypes of appendiceal tumors, SRCC has the worst prognosis, with a 3-year and 5-year survival rate of 39% and 18.4% respectively, and a median survival time of 26 months.^[1]^ Although with the progress of medical technology, the 5-year survival rate of SRCC is only 20.5%, and if there is metastasis, it will be reduced to 6.7% to 14%.^[32]^ Regardless of stage, right hemicolectomy was associated with better survival and prognosis than neither surgery nor appendectomy alone. Both preoperative neoadjuvant therapy and postoperative adjuvant chemoradiotherapy can reduce the recurrence rate and improve the survival rate, improve the prognosis, and bring clinical benefits to patients. Due to the high degree of malignancy, poor prognosis and common recurrence and metastasis of SRCC-A, long-term follow-up, dynamic monitoring of the disease changes and timely measures should be taken to improve the survival and prognosis of the patients.

## 4. Conclusion

SRCC-A is difficult to diagnose preoperatively or perioperatively due to its rare incidence and atypical clinical presentation. This case is a rare advanced stage patient diagnosed preoperatively. After receiving the FOLFOXIRI regimen combined with bevacizumab treatment, the patient showed a good curative response. Surgical treatment was performed after improvement of the clinical conditions, and adjuvant treatment was actively performed with the XELOX regimen to consolidate the curative effect. Thus, it is aggressive, and long-time follow-up is recommended for the evaluation of recurrence.

## Author contributions

**Conceptualization:** Nana Huang.

**Data curation:** Yishan Lu.

**Formal analysis:** Yishan Lu, Rui Wang.

**Methodology:** Rui Wang.

**Writing – original draft:** Nana Huang.

**Writing – review & editing:** Ping Gao, Ge Liu.
